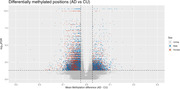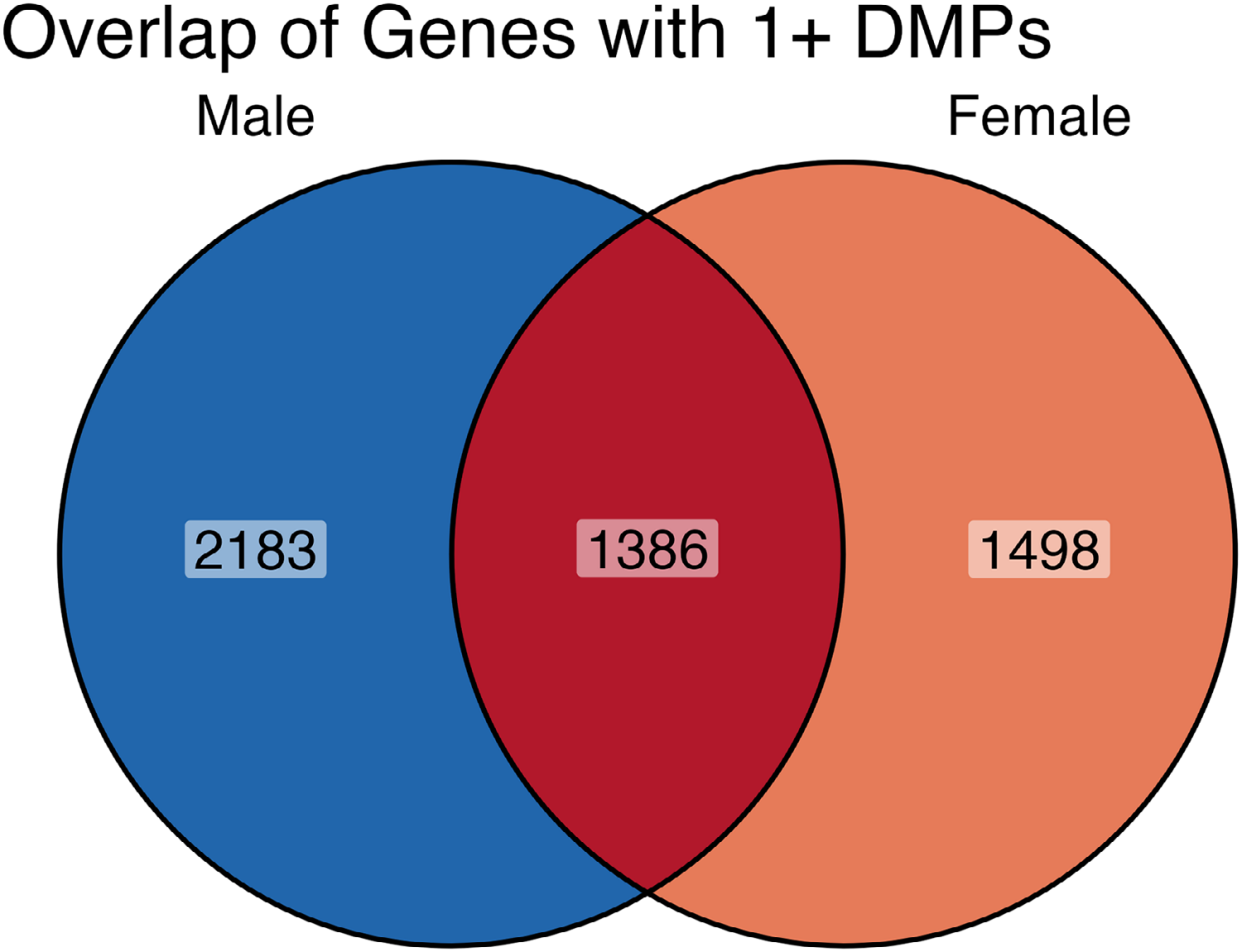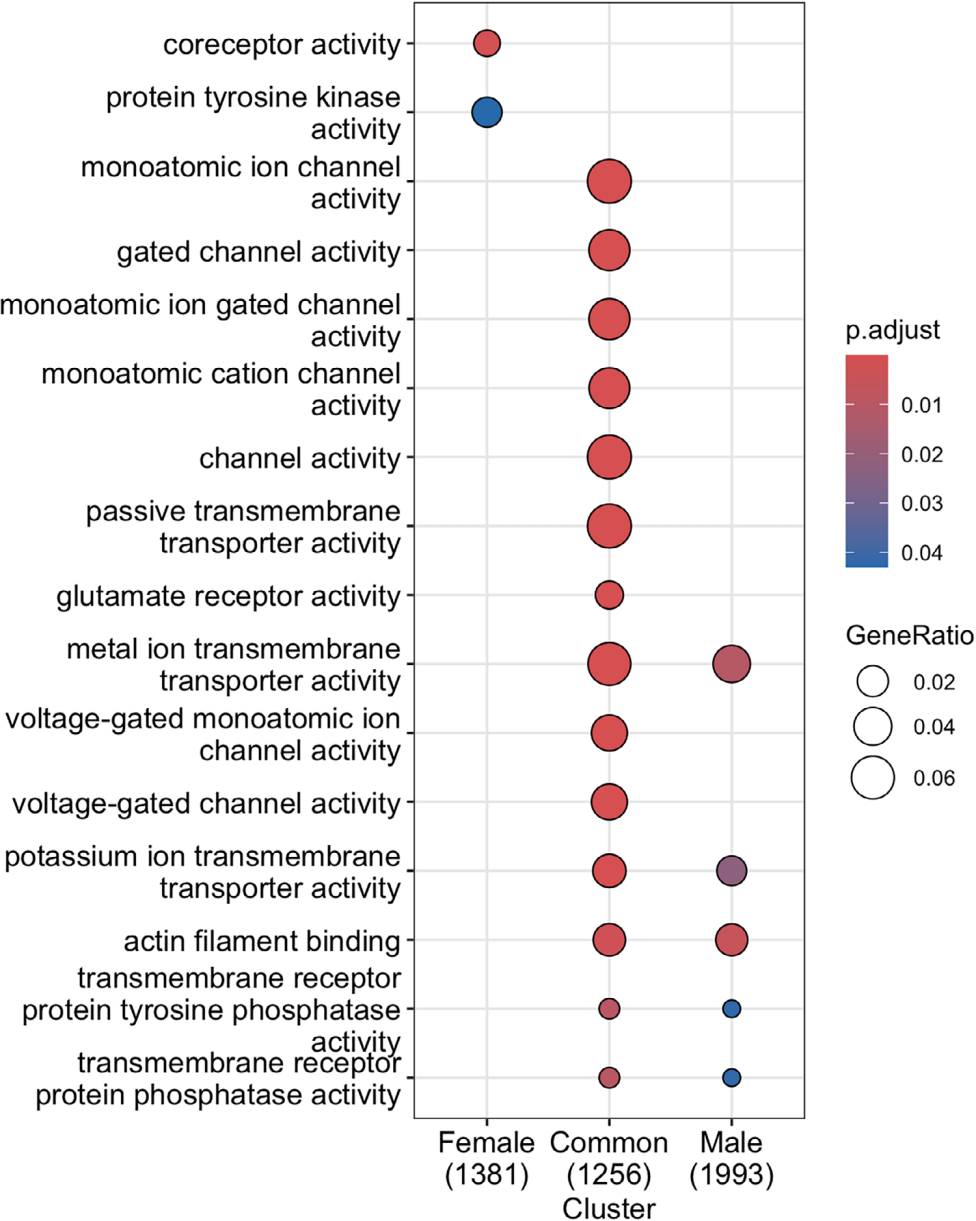# Whole genome methylation sequencing identifies sex‐specific DNA methylation levels in late onset dementia due to Alzheimer’s disease

**DOI:** 10.1002/alz.087422

**Published:** 2025-01-03

**Authors:** Phillip E Bergmann, Andy Madrid, Ligia A Papale, Coleman Breen, Lindsay R. Clark, Sanjay Asthana, Sterling C. Johnson, Sündüz Keles, Kirk J. Hogan, Reid S Alisch

**Affiliations:** ^1^ University of Wisconsin ‐ Madison, Madison, WI USA; ^2^ University of Wisconsin School of Medicine and Public Health, Madison, WI USA; ^3^ Geriatric Research Education and Clinical Center, William S. Middleton Memorial Veterans Hospital, Madison, WI USA; ^4^ Wisconsin Alzheimer’s Disease Research Center, University of Wisconsin School of Medicine and Public Health, Madison, WI USA; ^5^ Geriatric Research Education and Clinical Center William S. Middleton VA Hospital, Madison, WI USA; ^6^ Wisconsin Alzheimer’s Institute, University of Wisconsin School of Medicine and Public Health, Madison, WI USA; ^7^ Anesthesiology, University of Wisconsin School of Medicine and Public Health, Madison, WI USA; ^8^ University of Wisconsin‐Madison, Madison, WI USA

## Abstract

**Background:**

Late onset dementia due to Alzheimer’s disease (AD) has a sex‐biased incidence with females comprising nearly two thirds of all cases. Females have a more rapid progression in cognitive decline and higher levels of known AD biomarker pathology compared to men. Genetic sequence variation does not account for the sex‐biased incidence of AD, directing attention to the emerging role of epigenetics in AD. Using whole genome methylation sequencing (WGMS), we previously reported 28,038 differentially methylated position (DMPs) within 2,707 genes between persons with and without AD. Here we report sex‐specific DNA methylation levels in AD.

**Method:**

WGMS data quantified DNA methylation levels at 25,406,945 CpG loci in 281 blood samples from 109 AD (47 female, 62 male) and 174 cognitively unimpaired (CU) individuals (90 female, 84 male) in the Wisconsin Alzheimer’s Disease Research Center and the Wisconsin Registry for Alzheimer’s Prevention cohorts. Beta‐binomial regression models were developed to test for significant sex‐specific DNA methylation levels between CU and AD accounting for batch effects, estimated white blood cell proportions, BMI, and age. P‐values were corrected and local false discovery rates (lFDRs) were calculated.

**Result:**

13,293 DMPs were identified in females and 20,719 DMPs were identified in males (Figure 1) when comparing CU and AD (lFDR < 0.05; mean methylation difference > 2.5%). Approximately 2% (280) of the DMPs overlap between sexes with 87 having opposite mean methylation differences. The majority of DMPs annotate to introns (55.9% females; 54.8% males) with most of the remainder intergenic (37.0% females; 37.9% males). Annotation of DMPs to genes identified 1,498 genes that are only differentially methylated in females, and 2,183 genes that are only differentially methylated in males, with 1,365 differentially methylated genes shared between females and males (Figure 2). Gene ontological analyses identified significant enrichment of pathways related to glutamate receptor and ion channel activity in shared gene sets (Figure 3).

**Conclusion:**

33,732 unique, sex‐specific DMPs in AD were identified with more than 60% annotated to one of 5,067 genes. DMP‐associated genes have sex‐specific DNA methylation levels suggesting that these DMPs may play a role in the biased pathogenesis of AD.